# The Association of Fatty Acid Levels and Gleason Grade among Men Undergoing Radical Prostatectomy

**DOI:** 10.1371/journal.pone.0166594

**Published:** 2016-11-23

**Authors:** Zhiguo Zhao, Lael Reinstatler, Zachary Klaassen, Yi Xu, Xiaoyu Yang, Rabii Madi, Martha K. Terris, Steven Y. Qian, Uddhav Kelavkar, Kelvin A. Moses

**Affiliations:** 1 Department of Biostatistics, Vanderbilt-Ingram Cancer Center, Nashville, TN, USA; 2 Department of Surgery, Section of Urology, Medical College of Georgia–Augusta University, Augusta, Georgia, USA; 3 Department of Pharmaceutical Sciences, North Dakota State University, Fargo, ND, USA; 4 Nutechbiomarkers LLC, Savannah, Georgia, USA; 5 Department of Urologic Surgery, Vanderbilt University Medical Center, Nashville, Tennessee, USA; University of Minnesota Hormel Institute, UNITED STATES

## Abstract

**Background:**

Epidemiological data suggest that omega-6 (ω-6) fatty acids (FAs) may be associated with cancer incidence and/or cancer mortality, whereas ω-3 FAs are potentially protective. We examined the association of the ratio of ω-6 to ω-3 FA (ω-6:ω-3) and individual FA components with pathological results among men with prostate cancer (PCa) undergoing radical prostatectomy.

**Methods:**

Sixty-nine men were included in the study. Components of ω-6 (linoleic acid (LA), arachidonic acid (AA), and dihomo-γ-linolenic acid (DGLA)) and ω-3 (docosahexaenoic acid (DHA) and eicosapentaenoic acid (EPA)) were analyzed by liquid chromatography/mass selective detector separation. Logistic regression analysis was performed to determine association of FA with pathological high grade (Gleason ≥4+3) disease.

**Results:**

The were 35 men with low grade disease (Gleason ≤3+4) and 34 men with high grade disease. Men with low grade disease were significantly younger (58y vs 61y, p = 0.012) and had lower D’Amico clinical classification (p = 0.001) compared to men with high grade disease. There was no significant association of ω-6:ω-3 with high grade disease (OR 0.93, p = 0.78), however overall ω-6, ω-3, and individual components of ω-6 and ω-3 FAs except EPA were significantly associated with high grade disease (ω-6: OR 3.37, 95% CI: 1.27,8.98; LA: OR 3.33, 95% CI:1.24,8.94; AA: OR 2.93, 95% CI:1.24,6.94; DGLA: OR 3.21, 95% CI:1.28,8.04; ω-3: OR 3.47, 95% CI:1.22,9.83; DHA: OR 3.13, 95% CI:1.26,7.74). ω-6 and ω-3 FA components were highly correlated (Spearman ρ = 0.77).

**Conclusion:**

Higher levels of individual components of ω-6 and ω-3FAs may be associated with higher-grade PCa.

**Impact:**

Studies into the causative factors/pathways regarding FAs and prostate carcinogenesis may prove a potential association with PCa aggressiveness.

## Introduction

Prostate cancer (PCa) is the most common non-cutaneous malignancy in men and the second most common cause of cancer mortality.[1] With the advent of widespread prostate specific antigen (PSA) screening beginning in the early 1990s, there has been a significant stage migration at the time of diagnosis such that the majority of men are diagnosed with PCa at an earlier time point in the disease process.[2] Whether or not PSA screening has made a significant impact on cancer specific and overall mortality is a matter of debate, but it is well established that men can expect long-term cancer specific survival after treatment for localized disease.[3,4] A large percentage of men newly diagnosed with PCa have low risk disease, and may never become symptomatic or experience metastatic disease prior to death from other causes. In fact, recent data suggest that men with low grade disease (Gleason ≤ 6) will rarely experience metastatic disease.[5] Nevertheless, approximately one-third of men present with, or are at increased risk for, advanced disease and aggressive pathological features which places them at risk for biochemical recurrence and PCa mortality. The ability to differentiate those men who are at risk for aggressive disease from those who will have an indolent course remains elusive. The use of molecular markers or gene expression in serum or tissue is likely to be critical in this determination.

One specific area of interest in differentiating indolent from aggressive PCa is the role of fat intake and levels of fatty acids (FAs). Americans consume more processed plant fats and oils as compared to animal fats, and epidemiological studies indicate that this high-fat diet likely plays a role in increased risk of certain cancers. Specifically, consumption of ω-6 polyunsaturated FAs (PUFAs) correlates with increased risk for malignancy, whereas consumption of ω-3 FA, such as those found in fish oils, correlates with decreased risk of hepatocellular, colorectal and breast cancer. [6,7,8,9,10,11] In terms of PCa risk, epidemiological studies utilizing the Health Professionals Follow-Up Study and the Physicians’ Health Study indicated that increased intake of components of ω-6 FAs may be associated with more aggressive PCa, whereas greater consumption of ω-3 FAs may improve PCa survival.[12,13] Endogenous concentrations of ω-3 and ω-6 PUFAs directly reflect consumption since humans cannot synthesize these PUFAs de novo. Furthermore, while all mammalian cells can interconvert the PUFAs within each of the omega series, the two series are not interchangeable. Ideally, the ratio of ω-3 to ω-6 should be 1–4:1[14], but many Americans ingest 10 to 20 times more ω-6 than ω-3 FAs[15], leading to an imbalanced ratio[16,17], that potentially contributes to increased cancer incidence. Thus, PUFAs represent a promising target for disease impediment.

The distribution of FAs in red blood cell (RBC) membranes has been validated as an accurate estimate of the FAs in the diet. While there is a fair amount of in vitro and epidemiological data suggesting an association with imbalanced FAs, there are few data showing a direct correlation of FA levels and PCa severity. To address this deficit in knowledge, we measured ω-6 and ω-3 from RBC membranes of men undergoing radical prostatectomy for PCa to determine the association of FA levels and PCa aggressiveness. We hypothesized that elevated ω-6:ω-3 and/or higher levels of individual FA components are associated with higher grade (Gleason ≥4+3) disease.

## Materials and Methods

### Population

After obtaining Institutional Review Board (IRB) approval, men from a single institution (Medical College of Georgia Health Center) undergoing radical prostatectomy from December 2012 to July 2014 were recruited for participation in the study. Inclusion criteria included age greater than 35 years, appropriate surgical candidates with Gleason sum ≥6, clinical ≥T1c, and PSA ≥4ng/ml, and no prior history of malignancy except for adequately treated basal or squamous cell carcinomas of the skin or carcinoma “in situ” of the breast. None of the patients were taking 5-alpha reductase inhibitors. A total of 78 men underwent radical prostatectomy during the time period, of which 71 men met the inclusion criteria and provided serum samples. One sample was mailed incorrectly and one sample did not have ω-6 and ω-3 values reported, yielding a final study cohort of 69 samples. Levels of ω-6 (linoleic acid (LA), arachidonic acid (AA), and dihomo-γ-linolenic acid (DGLA)) and ω-3 (docosahexaenoic acid (DHA) and eicosapentaenoic acid (EPA)) FAs were extracted from membranes of RBCs obtained from fasting blood draws performed at the preoperative visit within one month of surgery. Demographic and clinical information collected from patients included age, self-reported race, body mass index (BMI), Charlson comorbidity index score (CCI), PSA, low-density lipoprotein (LDL), and biopsy results. Clinical and pathologic T(umor) classification was categorized according to the American Joint Committee on Cancer 7^th^ edition.

### Fatty Acid Extraction and Serum Measurements

FAs were extracted from washed erythrocyte membranes in the following manner. Whole blood was centrifuged at 2500 rpm for 20 minutes. The serum, buffy coat (containing white blood cells), and RBC layers were separated and labeled appropriately in tubes. An equal amount (about 1 ml) of chloroform, methanol and water in a 1:2:0.8 ratio was added to the RBC containing tube. Then, 100 μL of the RBC mixture was mixed with methanol and water to make a total of 3 mL of 15% methanol solution. Internal standards (PGE_2_-d_4_ and AA-d_8_) were added. The mixture was vortexed for 1 min, then set on ice for 30 min. After centrifuging for 15 min at 3000 rpm, the supernatant was collected and adjusted to pH 3.0. Then the sample solution was loaded on C-18 SPE cartridge column (preconditioned with 2 mL methanol and 2 mL water), washed with 1 mL water, and eluted with 2 mL ethyl acetate. The elution was condensed to dryness and reconstituted with 100 μL ethanol for LC/MS analysis.

High performance liquid chromatography selected reaction monitoring-mass spectrometry (LC SRM-MS) was performed for analysis and quantitation of the individual FAs using a triple quadrupole MS (TSQ ULTRA, Thermo Fisher Scientific, Waltham, MA, USA) equipped with an ultrapressure high performance liquid chromatographer (HPLC) (Accella, Thermo Fisher Scientific, Waltham, MA, USA). The LC/MS system consisted of an Agilent 1200 series HPLC system and an Agilent 6300 LC/MSD SL ion trap mass system. Separation was performed on a ZORBAX Eclipse XDB-C18 column (Agilent, 3.5 μm, 75 × 4.6 mm) at 25°C. Mobile Phase: A: 0.01% HOAc-H_2_O, B: 0.01% HOAc-ACN; Gradient: (i) 0–12 min (isocratic), 68% A and 32% B; (ii) 12–14 min, 68 to 44% A and 32 to 56% B; (iii) 14–28 min (isocratic), 44% A and 56% B; (iv) 28–30 min, 44 to 14% A and 56 to 86% B; (v) 30–38 min, 14 to 5% A and 86 to 95% B; and (vi) 38–44 min (isocratic), 5% A and 95% B. Flow Rate: 0.8 mL/min; Injection: 5 μL; Electrospray ionization in negative mode; Full scan from m/z 50 to m/z 500; Nebulizer Pressure: 15.0 psi; Dry Gas: 5.0 L/min; Dry Temperature: 325°C; Compound Stability: 20%; Number of scans: 50. The concentrations of PUFAs and PGs were quantified by comparing the ratios of the peak areas of the PUFAs and PGs to the internal standards. Individual FA analytes were quantified by SRM-MS, monitoring specific ion transitions, and normalized by reference to known isotope labeled standards that were used to spike samples prior to analysis (NuCheck Prep, Elysian, MN). The final results were normalized against the protein content of the original cellular extract to correct for variations in the amount of starting samples. LA (18:2, ω-6); GLA, γ-linoleic acid (18:3, ω-6); DGLA (20:3, ω-6); AA (20:4, ω-6); docosapentaenoic acid (22:5, ω-6); ALA, α-linoleic acid (18:3, ω-3); SDA, stearidonic acid (18:4, ω-3); EPA, eicosapentaenoic acid (20:5, ω-3); tetracosahexaenoic acid (24:6, ω-3); DPA, docosapentaenoic acid (22:5, ω-3), and DHA (22:6, ω-3) were analyzed and quantitated from the prostate tissues in the same manner as those from RBC membranes.[18] The ω-6:ω-3 FA ratio was reported as ω-6 (18:2 + 18:3 + 20:3 + 20:4 + 22:5) / ω-3 (18:3 + 18:4 + 20:5 + 24:6 + 22:5 + 22:6).[18]

### Statistical Analysis

Patients’ demographic characteristics, clinical information, and biomarker values were summarized by pathological Gleason grade (Low: Gleason ≤3+4 = 7 vs. High: Gleason ≥4+3 = 7) using median and interquartile range (IQR) for continues variables, or frequency with percentage for categorical variables. Differences between the two pathological Gleason groups were compared using the Wilcoxon rank sum test for continuous variables, or the χ^2^ test for categorical variables. We first assessed the correlations between ω-6 and ω-3, and among their components using Spearmen’s method. Given the fact that these components were highly correlated, we next assessed the associations between the ω-6: ω-3 ratio, ω-6, ω-3, and each individual FA component with pathological Gleason grade separately using multivariable logistic regression models. To prevent overfit of the model, patients’ age at biopsy and preoperative D’Amico classification were adjusted in all these models. All FAs were analyzed as continuous variables in all models. Odds ratios (OR) and 95% confidence intervals (95% CI) were estimated and reported. For all analyses, statistical significance was considered at a two-sided 5% level. No multiple comparisons adjustments were attempted. All analyses were conducted using R version 3.2.2.

## Results

Patient clinical and demographic information are summarized in Table 1 according to pathological Gleason sum. There were 35 patients with low grade (Gleason ≤3+4 = 7) disease and 34 patients with high grade (Gleason ≥4+3 = 7) disease. Men with low grade disease were significantly younger (58y vs 61y, p = 0.012), had lower comorbidity (CCI 3 vs 4, p = 0.006), and had significantly lower clinical disease characteristics (clinical Gleason sum, cT classification, preoperative PSA, and D’Amico classification).

**Table 1:** Patient Characteristics by Pathological Gleason Grade.

	Low Grade N = 35	High Grade N = 34	Combined N = 69	p-value
**Age (years)**	57.9 (51.8,60.7)	61.4 (55.4,65.4)	59.4 (53.7,63.9)	0.012^1^
**Race** ** • Black** ** • White**	60.0% (21) 40.0% (14)	58.8% (20) 41.2% (14)	59.4% (41) 40.6% (28)	0.92^2^
**BMI (kg/m**^**2**^**)**	29.7 (26.0,33.6)	30.4 (27.8,33.2)	30.0 (27.4,33.5)	0.76^1^
**CCI**	3 (2,4)	4 (3,5)	3 (3,4)	0.006^1^
**LDL (mg/dl)** ** • Missing Values = 27**	102.0 (76.5,118.5)	113.0 (83.0,128.0)	105.0 (81.0,123.5)	0.49^1^
**Clinical Gleason Sum** ** • Low (≤3+4)** ** • High (≥4+3)** ** • Missing Values = 4**	88.2% (30) 11.8% (4)	41.9% (13) 58.1% (18)	66.2% (43) 33.8% (22)	<0.001^2^
**Clinical T Classification** ** • T1** ** • T2** ** • Missing Values = 2**	94.1% (32) 5.9% (2)	66.7% (22) 33.3% (11)	80.6% (54) 19.4% (13)	0.005^2^
**PSA (ng/ml)** ** • Missing Values = 1**	5.27 (4.70,8.30)	8.95 (6.41,12.70)	6.85 (5.10,11.10)	0.002^1^
**D’Amico Classification** ** • Low** ** • Intermediate** ** • High**	42.9% (15) 51.4% (18) 5.7% (2)	14.7% (5) 47.1% (16) 38.2% (13)	29.0% (20) 49.3% (34) 21.7% (15)	0.001^2^
**ω-6:ω-3**	11.9 (7.3,23.0); 1.00	18.3 (9.6,26.0); 0.84	14.6 (7.7,24.7); 0.91	0.19^1^
**ω-6**	16.7 (3.0,28.4); 0.84	28.6 (19.0,48.6); 0.95	22.7 (9.8,40.7); 1.02	0.015^1^
**LA**	12.6 (2.9,21.9); 0.85	22.9 (15.8,38.5); 0.96	18.8 (8.1,33.8); 1.01	0.019^1^
**AA**	2.2 (0.3,4.6); 1.00	4.6 (1.9,10.1); 0.99	3.0 (0.9,6.0); 1.12	0.016^1^
**DGLA**	0.2 (0.04,0.4); 1.03	0.4 (0.2,0.9); 1.16	0.3 (0.09,0.6); 1.32	0.012^1^
**ω-3**	2.2 (0.2,3.0); 0.84	2.2 (0.9,3.6); 0.92	2.2 (0.3,3.3); 0.94	0.23^1^
**DHA**	0.4 (0.1,1.1); 1.07	1.0 (0.6,2.0); 1.13	0.7 (0.2,1.5); 1.23	0.01^1^
**EPA**	0.8 (0.06,2.1); 1.05	0.8 (0.2,1.6); 1.38	0.8 (0.08,1.9); 1.24	0.95^1^

Low Grade- Gleason 6, 7 (3+4), High Grade- Gleason 7 (4+3), 8–10.

AA- arachidonic acid, BMI-body mass index, CCI- age-adjusted Charlson comorbidity index, DGLA- dihomo- γ-linoleic acid, DHA- docosahexaenoic acid, EPA- eicosapentaenoic acid, IQR-interquartile range, LA- linoleic acid, LDL-low density lipoprotein, PSA- prostate specific antigen, T- tumor.

Values are median (25%, 75% quartiles); coefficient of variance. Numbers after percents are frequencies.

Tests used

^1^ Wilcoxon test

^2^ Pearson test.

The ratio of ω-6 to ω-3 (ω-6:ω-3) FA was not found to be significantly different between the low grade and high grade groups (p = 0.19). However, median levels of ω-6 FAs overall, and the individual components of ω-6, were significantly lower in the low vs high grade patients (ω-6: 16.7 (3.0,28.4) vs 28.6 (19.0,48.6), p = 0.015; LA: 12.6 (2.9,21.9) vs 22.9 (15.8,38.5), p = 0.019; AA: 2.2 (0.3,4.6) vs 4.6 (1.9,10.1), p = 0.016; DGLA: 0.2 (0.04,0.4) vs 0.4 (0.2,0.9), p = 0.012). There was no difference in the median overall ω-3 FAs between the two groups (p = 0.23), or in the ω-3 subcomponent EPA (p = 0.95); however, median DHA was significantly lower in the low grade group (DHA: 0.4 (0.1,1.1) vs 1.0 (0.6,2.0), p = 0.01).

We performed Spearman correlation analysis of the FAs to examine the feasibility of looking at them separately vs as a group. ω-6 and ω-3 are highly correlated (Spearman ρ = 0.77, p <0.001). The individual ω-6 and ω-3 FA components were also highly correlated (summarized in Table 2, all pairwise comparisons p<0.001).

**Table 2:** Spearman Correlation Coefficients of Individual ω-6 and ω-3 Components.

	**LA**	**AA**	**DGLA**	**DHA**	**EPA**
**LA**	-	0.89	0.93	0.89	0.51
**AA**	0.89	-	0.96	0.92	0.62
**DGLA**	0.93	0.96	-	0.90	0.58
**DHA**	0.89	0.92	0.90	-	0.51
**EPA**	0.51	0.62	0.58	0.51	-

All pairwise comparisons p<0.001.

AA- arachidonic acid, DGLA- dihomo- γ-linoleic acid, DHA- docosahexaenoic acid, EPA- eicosapentaenoic acid, LA- linoleic acid.

We performed logistic regression analysis to estimate the ORs of having high grade disease (Gleason ≥4+3) on pathological specimen comparing the upper (75%) quartile to the lower (25%) quartile of FA components, adjusting for age and D’Amico classification (Table 3). ω-6:ω-3 was not found to be associated with high grade disease (OR 0.93, p = 0.78); however, total ω-6 was significantly associated with higher odds of high grade disease (OR 3.37, 95% CI:1.27,8.98, p = 0.015), as were the individual ω-6 FA components (LA: OR 3.33, 95% CI:1.24,8.94, p = 0.017; AA: OR 2.93, 95% CI:1.24,6.94, p = 0.014; DGLA: OR 3.21, 95% CI:1.28,8.04, p = 0.013). Total ω-3 was also significantly associated with high grade disease (OR 3.47, 95% CI:1.22,9.83, p = 0.019). The DHA component demonstrated increased likelihood of high grade disease (OR 3.13, 95% CI:1.26,7.74, p = 0.014), but EPA did not (OR 1.57, p = 0.22).

**Table 3:** Odds Ratio (95% Confidence Interval) of Pathological High Grade Disease (≥4+3).

	**OR (95% CI)**	**p-value**
**ω-6:ω-3**	0.93 (0.56,1.54)	0.78
**ω-6**	3.37 (1.27,8.98)	0.015
** • LA**	3.33 (1.24,8.94)	0.017
** • AA**	2.93 (1.24,6.94)	0.014
** • DGLA**	3.21 (1.28,8.04)	0.013
**ω-3**	3.47 (1.22,9.83)	0.019
** • DHA** ** • EPA**	3.13 (1.26,7.74)	0.014
	1.57 (0.76,3.22)	0.22

Logistic regression analysis adjusted for age and D’Amico classification. All ORs are comparing upper quartile (Q3) to lower quartile (Q1).

AA- arachidonic acid, DGLA- dihomo- γ-linoleic acid, DHA- docosahexaenoic acid, EPA- eicosapentaenoic acid, LA- linoleic acid.

## Discussion

In this study, we sought to explore the potential association of FA with PCa among men undergoing radical prostatectomy through examining RBC membrane FA concentrations. Specifically, we sought to examine the association of ω-6:ω-3, as well as the individual components of ω-6 and ω-3 FAs with Gleason sum, as these are shown in animal studies to be associated with increased and decreased risk for PCa, respectively.[19,20] We demonstrate that elevated ω-6 and ω-3 FAs are significantly associated with high grade PCa, except for the ω-3 FA EPA. Additionally, we found that the individual FAs are highly correlated, likely explaining the non-significant association of ω-6:ω-3 in this cohort of patients. This is one of the first studies showing an association of both ω-6 and ω-3 FAs with PCa aggressiveness not based on epidemiological studies or in vitro/in vivo models, and suggests that FA concentrations may have an effect on PCa grade.

In vitro and animal studies suggest that the metabolites of PUFAs directly impact PCa and that the ability to do so depends on both diet and metabolic enzyme expression.[21,22,23,24,25,26,27,28,29,30] There is elevated expression of 15-lipoxygenase-1 (15-LO-1) in prostate tumor tissue as compared with normal adjacent tissue.[29] Human 15-LO-1 is a highly-regulated, tissue- and cell-type specific lipid-peroxidating enzyme that has several functions ranging from physiological membrane remodeling to pathogenesis of atherosclerosis, inflammation and carcinogenesis. 15-LO-1 metabolizes LA to 13(S)-hydroxyoctadecadienoic acid [13-(S)-HODE], which can regulate cell growth, differentiation and vascular homeostasis. Data from the Kelavkar laboratory has shown that 13-(S)-HODE is mitogenic and enhances cellular proliferation.[21,28,29,30] Moreover, EPA successfully competes with LA as a substrate for 15-LO-1, resulting in a decrease in the pro-tumorigenic metabolite of LA, 13(S)-HODE, and in increase in the anti-tumorigenic metabolite if EPA, 15-hydroxyeicosapentaenoic acid (15-HEPE)[31,32]; and EPA competes successfully with AA for COX-2 activity. In vitro studies have indeed shown that inhibition of 15-LO-1 enzyme activity causes apoptosis in the PCa cell line PC3.[28,29] Dietary manipulation in mice has confirmed that an ω-3 diet modulated PSA levels, promoted apoptosis and inhibited proliferation in tumors in a xenograft mouse model.[18] This study in the mouse model confirmed the earlier findings that: 1) EPA successfully competes with LA as a substrate for 15-LO-1, resulting in a decrease in pro-tumorigenic 13(S)HODE and an increase in the anti-tumorigenic 15-HEPE; and 2) EPA competes successfully with AA for COX-2 activity, suggesting that there may be a causal link between dietary FAs and PCa.

Most of the attention in the scientific and lay press regarding the benefits and risks of PCa screening in men centers on the necessity of diagnosis or treatment for potentially indolent disease. However, data indicate that certain populations of men at intermediate or high risk may derive benefit from treatment of PCa.[33] Because PSA and DRE are not highly sensitive tests, there is no threshold below which men will not be diagnosed with PCa, and there is currently no test that can accurately predict whether men with low risk disease (i.e.- Gleason 3+3 = 6, cT1c or cT2a, and PSA <10ng/ml) will continue on an indolent course or progress to more aggressive disease. Therefore, a safe and easily reproducible test that can identify those at risk for aggressive disease will obviate concerns of harms from screening and treatment, and men can be managed in a more standardized and appropriate manner. Additionally, if a strong association exists between membrane FA levels and PCa grade or stage, dietary manipulation of ω-3 FA may serve as a way of reducing the incidence of, or slowing the progression of cancer in men.

There are some limitations to consider for this study. As this was an exploratory study, our patient cohort is small, and therefore results may not be generalizable until a larger, confirmatory study can be performed. This is one potential explanation why there was no significant association of ω-6:ω-3 and pathological Gleason grade. With a sample size of 69, our study has 80% power to detect an OR of 2 or greater (or 0.5 or smaller) for every 1 standard deviation increase in FAs while maintains the type I error rate at 0.05. The minimum ω-6:ω-3 ratio of 4.3 was still well above the previously defined normal ratio (~1). Therefore, a greater range of ω-6:ω-3 ratios, in addition to inclusion of men with benign and locally aggressive/metastatic disease, may add generalizability to the findings presented here. Second, the current data is based solely on pathological data. Long term follow-up to determine the potential association of FA concentrations with biochemical recurrence or development of metastatic disease will be focus of future investigation. An example of this is demonstrated in recent data from the SEARCH database suggesting an association between elevated serum triglycerides and PCa recurrence after radical prostatectomy [34]. Third, as discussed in a recent study by Yang et. al, the timing of blood samples collection may play a role of associations between the FAs and prostate cancer.[35] All our study samples were collected within 1 month of the radical prostatectomy, thus there was no strong evidence that the observed associations lead to a causation. Overall, our preliminary data suggest a potential association that merits further exploration on a larger scale, ideally a randomized controlled trial. The ability of FA concentrations compared to PSA and/or other methods of detecting clinically significant cancer should be examined.

## References

[1] Siegel R, Naishadham D, Jemal A (2012) Cancer statistics, 2012. CA Cancer J Clin 62: 10–29. 10.3322/caac.20138 22237781

[2] Brawley OW (2012) Prostate cancer epidemiology in the United States. World J Urol 30: 195–200. 10.1007/s00345-012-0824-2 22476558

[3] Loeb S, Schaeffer EM, Trock BJ, Epstein JI, Humphreys EB, et al (2010) What are the outcomes of radical prostatectomy for high-risk prostate cancer? Urology 76: 710–714. 10.1016/j.urology.2009.09.014 19931898 PMC2889156

[4] Dorin RP, Daneshmand S, Lassoff MA, Cai J, Skinner DG, et al (2012) Long-term outcomes of open radical retropubic prostatectomy for clinically localized prostate cancer in the prostate-specific antigen era. Urology 79: 626–631. 10.1016/j.urology.2011.09.051 22245303

[5] Kweldam CF, Wildhagen MF, Bangma CH, van Leenders GJ (2014) Disease-specific death and metastasis do not occur in patients with Gleason score </ = 6 at radical prostatectomy. BJU Int. 10.1111/bju.12879 25060593

[6] Abel S, Riedel S, Gelderblom WC (2014) Dietary PUFA and cancer. Proc Nutr Soc 73: 361–367. 10.1017/S0029665114000585 24850051

[7] Lawrence GD (2013) Dietary fats and health: dietary recommendations in the context of scientific evidence. Adv Nutr 4: 294–302. 10.3945/an.113.003657 23674795 PMC3650498

[8] Sawada N, Inoue M, Iwasaki M, Sasazuki S, Shimazu T, et al (2012) Consumption of n-3 fatty acids and fish reduces risk of hepatocellular carcinoma. Gastroenterology 142: 1468–1475. 10.1053/j.gastro.2012.02.018 22342990

[9] Sasazuki S, Inoue M, Iwasaki M, Sawada N, Shimazu T, et al (2011) Intake of n-3 and n-6 polyunsaturated fatty acids and development of colorectal cancer by subsite: Japan Public Health Center-based prospective study. Int J Cancer 129: 1718–1729. 10.1002/ijc.25802 21120874

[10] Hall MN, Chavarro JE, Lee IM, Willett WC, Ma J (2008) A 22-year prospective study of fish, n-3 fatty acid intake, and colorectal cancer risk in men. Cancer Epidemiol Biomarkers Prev 17: 1136–1143. 10.1158/1055-9965.EPI-07-2803 18483335 PMC3681614

[11] Kuriki K, Hirose K, Wakai K, Matsuo K, Ito H, et al (2007) Breast cancer risk and erythrocyte compositions of n-3 highly unsaturated fatty acids in Japanese. Int J Cancer 121: 377–385. 10.1002/ijc.22682 17354239

[12] Chavarro JE, Stampfer MJ, Hall MN, Sesso HD, Ma J (2008) A 22-y prospective study of fish intake in relation to prostate cancer incidence and mortality. Am J Clin Nutr 88: 1297–1303. 18996866 10.3945/ajcn.2008.26419 PMC2843087

[13] Leitzmann MF, Stampfer MJ, Michaud DS, Augustsson K, Colditz GC, et al (2004) Dietary intake of n-3 and n-6 fatty acids and the risk of prostate cancer. Am J Clin Nutr 80: 204–216. 15213050 10.1093/ajcn/80.1.204

[14] Simopoulos AP, Leaf A, Salem N Jr. (1999) Essentiality of and recommended dietary intakes for omega-6 and omega-3 fatty acids. Ann Nutr Metab 43: 127–130. 10436312 10.1159/000012777

[15] Allison DB, Egan SK, Barraj LM, Caughman C, Infante M, et al (1999) Estimated intakes of trans fatty and other fatty acids in the US population. J Am Diet Assoc 99: 166–174; quiz 175–166. 10.1016/S0002-8223(99)00041-3 9972183

[16] Dunn JE (1975) Cancer epidemiology in populations of the United States—with emphasis on Hawaii and California—and Japan. Cancer Res 35: 3240–3245. 1192400

[17] Haenszel W, Kurihara M (1968) Studies of Japanese migrants. I. Mortality from cancer and other diseases among Japanese in the United States. J Natl Cancer Inst 40: 43–68. 5635018

[18] Kelavkar UP, Hutzley J, Dhir R, Kim P, Allen KG, et al (2006) Prostate tumor growth and recurrence can be modulated by the omega-6:omega-3 ratio in diet: athymic mouse xenograft model simulating radical prostatectomy. Neoplasia 8: 112–124. 10.1593/neo.05637 16611404 PMC1578514

[19] Rose DP, Connolly JM (1999) Omega-3 fatty acids as cancer chemopreventive agents. Pharmacol Ther 83: 217–244. 10576293 10.1016/s0163-7258(99)00026-1

[20] Connolly JM, Coleman M, Rose DP (1997) Effects of dietary fatty acids on DU145 human prostate cancer cell growth in athymic nude mice. Nutr Cancer 29: 114–119. 10.1080/01635589709514611 9427973

[21] Kelavkar UP, Cohen C, Kamitani H, Eling TE, Badr KF (2000) Concordant induction of 15-lipoxygenase-1 and mutant p53 expression in human prostate adenocarcinoma: correlation with Gleason staging. Carcinogenesis 21: 1777–1787. 11023533 10.1093/carcin/21.10.1777

[22] Liu B, Khan WA, Hannun YA, Timar J, Taylor JD, et al (1995) 12(S)-hydroxyeicosatetraenoic acid and 13(S)-hydroxyoctadecadienoic acid regulation of protein kinase C-alpha in melanoma cells: role of receptor-mediated hydrolysis of inositol phospholipids. Proc Natl Acad Sci U S A 92: 9323–9327. 7568126 10.1073/pnas.92.20.9323 PMC40977

[23] Tang DG, Honn KV (1994) 12-Lipoxygenase, 12(S)-HETE, and cancer metastasis. Ann N Y Acad Sci 744: 199–215. 7825842 10.1111/j.1749-6632.1994.tb52738.x

[24] Honn KV, Tang DG, Grossi I, Duniec ZM, Timar J, et al (1994) Tumor cell-derived 12(S)-hydroxyeicosatetraenoic acid induces microvascular endothelial cell retraction. Cancer Res 54: 565–574. 8275495

[25] Chen YQ, Duniec ZM, Liu B, Hagmann W, Gao X, et al (1994) Endogenous 12(S)-HETE production by tumor cells and its role in metastasis. Cancer Res 54: 1574–1579. 7511046

[26] Nie D, Hillman GG, Geddes T, Tang K, Pierson C, et al (1998) Platelet-type 12-lipoxygenase in a human prostate carcinoma stimulates angiogenesis and tumor growth. Cancer Res 58: 4047–4051. 9751607

[27] Ghosh J, Myers CE (1998) Inhibition of arachidonate 5-lipoxygenase triggers massive apoptosis in human prostate cancer cells. Proc Natl Acad Sci U S A 95: 13182–13187. 9789062 10.1073/pnas.95.22.13182 PMC23752

[28] Kelavkar U, Glasgow W, Eling TE (2002) The effect of 15-lipoxygenase-1 expression on cancer cells. Curr Urol Rep 3: 207–214. 12084190 10.1007/s11934-002-0066-8

[29] Kelavkar UP, Nixon JB, Cohen C, Dillehay D, Eling TE, et al (2001) Overexpression of 15-lipoxygenase-1 in PC-3 human prostate cancer cells increases tumorigenesis. Carcinogenesis 22: 1765–1773. 11698337 10.1093/carcin/22.11.1765

[30] Kelavkar UP, Cohen C (2004) 15-lipoxygenase-1 expression upregulates and activates insulin-like growth factor-1 receptor in prostate cancer cells. Neoplasia 6: 41–52. 15068670 10.1016/s1476-5586(04)80052-6 PMC1508629

[31] Narayanan NK, Narayanan BA, Reddy BS (2005) A combination of docosahexaenoic acid and celecoxib prevents prostate cancer cell growth in vitro and is associated with modulation of nuclear factor-kappaB, and steroid hormone receptors. Int J Oncol 26: 785–792. 15703837

[32] Pham H, Ziboh VA (2002) 5 alpha-reductase-catalyzed conversion of testosterone to dihydrotestosterone is increased in prostatic adenocarcinoma cells: suppression by 15-lipoxygenase metabolites of gamma-linolenic and eicosapentaenoic acids. J Steroid Biochem Mol Biol 82: 393–400. 12589947 10.1016/s0960-0760(02)00217-0

[33] Wilt TJ, Brawer MK, Jones KM, Barry MJ, Aronson WJ, et al (2012) Radical prostatectomy versus observation for localized prostate cancer. N Engl J Med 367: 203–213. 10.1056/NEJMoa1113162 22808955 PMC3429335

[34] Allott EH, Howard LE, Cooperberg MR, Kane CJ, Aronson WJ, et al (2014) Serum lipid profile and risk of prostate cancer recurrence: Results from the SEARCH database. Cancer Epidemiol Biomarkers Prev 23: 2349–2356. 10.1158/1055-9965.EPI-14-0458 25304929 PMC4221498

[35] Yang M, Sesso HD, Colditz GA, Ma J, Stampfer MJ, et al (2016) Effect Modification by Time Since Blood Draw on the Association Between Circulating Fatty Acids and Prostate Cancer Risk. J Natl Cancer Inst 108. 10.1093/jnci/djw141 PMC5241906 27297429

